# The Effect of Pulling Angle on Rotator Cuff Mechanical Properties in a Canine In Vitro Model

**DOI:** 10.3390/bioengineering10050599

**Published:** 2023-05-17

**Authors:** Qian Liu, Jun Qi, Weihong Zhu, Andrew R. Thoreson, Kai-Nan An, Scott P. Steinmann, Chunfeng Zhao

**Affiliations:** 1 Department of Orthopaedics, The Second Xiangya Hospital, Central South University, Changsha 410011, China; 2 Department of Orthopedics, Tongji Hospital, Huazhong University of Science and Technology, Wuhan 430030, China; 3 Department of Orthopedic Surgery, Mayo Clinic, Rochester, MN 55905, USA; 4 Department of Orthopedic Surgery, University of Tennessee Health Science Center College of Medicine, Chattanooga, TN 37450, USA

**Keywords:** rotator cuff, canine model, biomechanics, biomechanical testing, pulling angle

## Abstract

The objective of this study was to examine the effect of pulling angle on time-zero mechanical properties of intact infraspinatus tendon or infraspinatus tendon repaired with the modified Mason-Allen technique in a canine model in vitro. Thirty-six canine shoulder samples were used. Twenty intact samples were randomly allocated into functional pull (135°) and anatomic pull (70°) groups (*n* = 10 per group). The remaining sixteen infraspinatus tendons were transected from the insertion and repaired using the modified Mason-Allen technique before being randomly allocated into functional pull or anatomic pull groups (*n* = 8 per group). Load to failure testing was performed on all specimens. The ultimate failure load and ultimate stress of the functional pulled intact tendons were significantly lower compared with anatomic pulled tendons (1310.2 ± 167.6 N vs. 1687.4 ± 228.2 N, *p* = 0.0005: 55.6 ± 8.4 MPa vs. 67.1 ± 13.3 MPa, *p* = 0.0334). For the tendons repaired with the modified Mason-Allen technique, no significant differences were observed in ultimate failure load, ultimate stress or stiffness between functional pull and anatomic pull groups. The variance of pulling angle had a significant influence on the biomechanical properties of the rotator cuff tendon in a canine shoulder model in vitro. Load to failure of the intact infraspinatus tendon was lower at the functional pulling position compared to the anatomic pulling position. This result indicates that uneven load distribution across tendon fibers under functional pull may predispose the tendon to tear. However, this mechanical character is not presented after rotator cuff repair using the modified Mason-Allen technique.

## 1. Introduction

Rotator cuff injury is the most common cause of shoulder dysfunction [1], with a prevalence of nearly 13% in people older than 50 years of age [2]. Although surgical repair of torn rotator cuff has shown satisfactory results with pain relief and functional improvement, the postoperative retear rate is reported to range from 15% to 94% [3,4,5,6,7]. There are a variety of factors affecting the healing of repaired rotator cuff which involve surgical techniques, rehabilitation protocols, size of tear, tissue quality, comorbidities and smoking [8]. Using magnetic resonance imaging or computed tomographic arthrography, Park et al. reported a significantly higher postoperative failure rate in patients who have a tear size larger than 2 cm compared to those with a smaller tear at a minimum one-year follow-up. They also found a positive correlation between Goutallier grade II or higher muscle fatty degeneration and increased failure rate [9]. Another cohort study demonstrated 96% healing in small or medium tears compared with 78% healing in large or massive tears after arthroscopic rotator cuff repair at a minimum of 2 years of follow-up [10]. Through collecting muscle samples from heavy smokers and matched non-smokers during arthroscopic repair of medium-sized rotator cuff tears, Lee et al. identified a series of genes with upregulated expression that regulates inflammation, fatty degeneration and fibrosis, such as high mobility group box 1 (HMGB1), peroxisome proliferator-activated receptor gamma (PPARγ) and fibrogenic alpha-smooth muscle actin (α-SMA). The histological observation further corroborated the gene expression patterns that increased inflammatory cell infiltration, fatty area and fibrogenic area were seen in rotator cuff muscles from smokers compared to that in non-smokers [11].

To improve the postoperative outcomes of rotator cuff repair, a better understanding of the pathophysiology of rotator cuff tear and the exploration of more effective therapeutic strategies is required. Currently, a number of animals that can model the features of human rotator cuff, including the anatomy, biomechanical and biological properties, have been established for the evaluation of novel therapies [12]. Animal models of rotator cuff injury can generally be categorized into small animal models, such as rat and mouse, or large animal models, such as sheep, goat, dog and nonhuman primate. Considering the closer anatomical structure to humans and the ease of conducting clinically relevant approaches, large animals are critical tools that bridge preclinical studies and clinical trials, playing an indispensable role in investigating the efficacy of newly developed treatment strategies for rotator cuff injury prior to potential clinical translation [13]. In addition to histological evaluation that mainly focuses on the cell infiltration, vascularity, diameter and orientation of collagen fibers, and the formation of fibrocartilage, biomechanical testing serves as a primary outcome to assess the initial strength and healing quality of the repaired tendon. It is estimated that biomechanical testing has been performed in more than 50% of studies on rotator cuff injury and repair using animal models [12]. Regaining a similar maximum load to failure, stiffness and maximum stress to the normal tendon is the aim of every tested repair method. However, the positioning of animal shoulder specimens during biomechanical testing, specifically the angle between the humeral shaft and the cuff tendon, varies among studies.

The most commonly used rotator cuff tendons in animal models are the supraspinatus and infraspinatus tendons [14,15,16,17,18,19,20]. While the majority of studies pulled the tendon along its anatomic vector [21,22,23,24,25,26] or at 90° abduction, mimicking a mid-stance phase during animal walking [17,27,28], several studies performed the biomechanical testing at a functional position to simulate a heel-strike phase during animal walking [15,16,29,30,31]. It is noteworthy that only a few studies specified the pulling angle between the cuff tendon and humeral shaft during biomechanical testing. Nicholson et al. set a 105° angle between the infraspinatus tendon and humeral shaft for biomechanical testing in an ovine model [26]. Rossbach et al. aligned the infraspinatus tendon to the humeral shaft at a 90° angle in a sheep model [29]. Furthermore, biomechanical testing could be done in either an anatomic or functional direction of pull in the same animal model [23,29]. Thus, it seems that there has been no consensus regarding the pulling angle for evaluating rotator cuff mechanical properties in different animal models, which would make the interpretation and comparison of testing results between studies difficult. Although Newton et al. have demonstrated that the biomechanical properties of the rat supraspinatus tendon changed significantly as a result of the variance of abduction testing angle [32], it remains unclear whether this phenomenon would also present in the infraspinatus tendon, especially for preclinical large animal models.

Canine shoulder is one of the most widely employed models for preclinical rotator cuff-related research. Compared to other large animals, such as cows, goats or sheep, the advantages of the canine shoulder model are a high tolerance of various postoperative rehabilitation protocols and similarity to humans in terms of rotator cuff muscle architecture and joint mechanical environment [14,33]. In previous studies, the authors have successfully utilized the canine shoulder model to evaluate novel surgical techniques or tissue engineering strategies for improving rotator cuff repair and regeneration [16,30,31]. The configuration of suture has a significant effect on repair strength. Specifically, the repair strength is closely associated with the suture materials, the number of strands and the area of suture-tendon interface. It has been shown that the arthroscopic modified Mason-Allen technique contributed to footprint restoration and satisfactory postoperative outcomes in patients with large U- or L-shaped cuff tears at a minimum two-year follow-up [34]. Compared to suture-bridge repair, Lee et al. also found comparable clinical and radiologic outcomes of patients with full-thickness cuff tears who underwent arthroscopic double-row modified Mason-Allen repair [35]. In addition, the modified Mason-Allen technique is a preferred repair method for canine rotator cuff tendons, given the merits of sufficient repair strength and well tissue preservation [21,22]. Therefore, the purpose of this study was to examine the effect of pulling angle on time-zero mechanical properties of an intact infraspinatus tendon or an infraspinatus tendon repaired with the modified Mason-Allen technique in a canine model in vitro. We hypothesized that the intact infraspinatus tendon pulled at the functional angle would demonstrate lower failure strength than that at the anatomic angle, and the modified Mason-Allen repair would overcome the effect of pulling angle and yield similar mechanical strength.

## 2. Materials and Methods

### 2.1. Experimental Design

**Figure 1:**
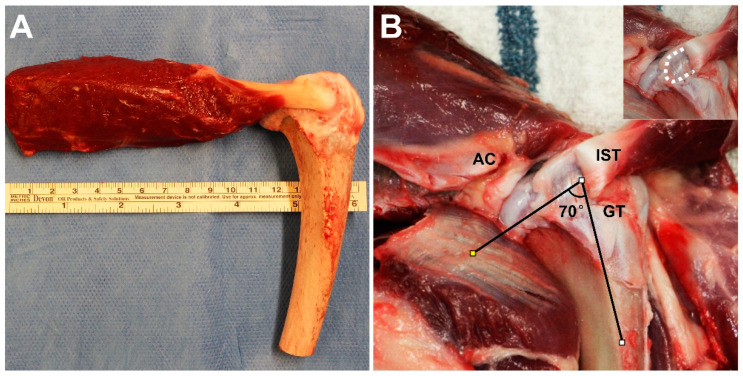
(**A**) A photograph of the dissected muscle-tendon-bone complex. (**B**) The determination of the anatomic pull angle of 70°. The concavity on the caudal aspect of the greater tubercle through which the infraspinatus tendon passes was indicated with the white dashed line (insert). AC, acromion; IST, infraspinatus tendon; GT, greater tubercle.

Thirty-six canine shoulders were harvested from mixed-breed dogs (body weight 24 ± 2 kg) euthanized for other studies approved by our Institutional Animal Care and Use Committee (IACUC No. A15815-15). A length of the infraspinatus muscle approximately 10 cm long was detached from the fossa infraspinata, along with its tendon attachment on the humerus with complete removal of irrelevant soft tissues (Figure 1A). The muscle-tendon-bone complex was then wrapped in saline-soaked gauze and stored at −20 °C until biomechanical testing. Twenty intact samples were randomly allocated into two groups (*n* = 10 per group): functional pull (135°) and anatomic pull (70°). The remaining sixteen infraspinatus tendons were repaired with the modified Mason-Allen technique and were also randomly allocated into functional pull or anatomic pull groups (*n* = 8 per group). The 135° angle of functional pull was based on previous studies [16,30,36]. To determine the anatomic pull angle, the authors found there is a concavity on the caudal aspect of the greater tubercle through which the infraspinatus tendon passes. The angle between the concavity and humeral longitudinal vector was consequently measured to represent the anatomic direction of pull (Figure 1B). A power analysis was performed based on previous studies [30,36], which showed eight samples per group was enough to reach a statistical power of 80% with α level of 0.05 for biomechanical testing.

### 2.2. Rotator Cuff Repair

The canine infraspinatus tendon was sharply transected at full-width from its insertion on the greater tubercle of the humerus using a scalpel blade. Two transosseous bone tunnels were subsequently created at the greater tubercle with a 1-mm-diameter drill bit. After securing the infraspinatus tendon to its insertion via the modified Mason-Allen technique with two 0-Fiberwire sutures (Arthrex, Naples, FL, USA) passed through the transosseous tunnels, the sutures were tied over the lateral aspect of the humerus.

### 2.3. Biomechanical Testing

**Figure 2:**
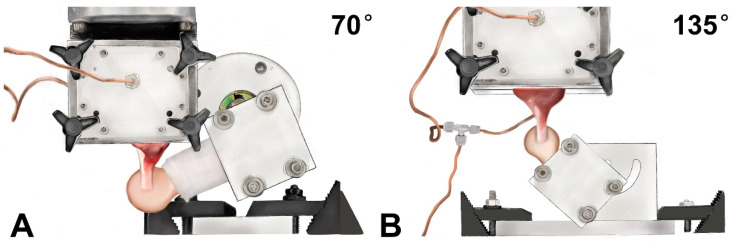
The positioning of canine shoulder specimens during biomechanical testing simulating (**A**) anatomic pull and (**B**) functional pull.

A day prior to biomechanical testing, the samples were thawed at room temperature. The cross-sectional area of the intact or repaired infraspinatus tendons was estimated at the insertion site using a digital caliper, assuming a rectangular shape. Biomechanical testing was performed using a servohydraulic materials testing system (MTS 312, MTS Systems Corp, Minneapolis, MN, USA). The infraspinatus muscle belly was gripped by a custom cryoclamp cooled by liquid carbon dioxide to prevent muscle damage and slippage during the testing. The humeral shaft of each specimen was potted in an aluminum tube with polymethylmethacrylate. After each specimen was fixed to the testing machine, a 70° or 135° angle between the longitudinal axis of the infraspinatus tendon and the humeral shaft was set to simulate anatomic and functional pull, respectively (Figure 2). Load to failure testing was subsequently conducted at a rate of 30 mm/min in accordance with previous studies [16,30]. The ultimate failure load was determined as the peak force obtained throughout the testing. Failure modes for all specimens were recorded. The ultimate stress was calculated by dividing the ultimate failure load with the initial cross-sectional area. The repair stiffness was calculated from the linear region of the load-displacement curve. Mechanical testing was performed at room temperature, and saline solution spray was used to keep specimens moist.

### 2.4. Statistical Analysis

Numerical data are presented as mean and standard deviation, and were tested for normality and equal variance before statistical analysis. The comparison of ultimate failure load and ultimate stress of the intact tendons between groups was performed using the unpaired Student *t* test. The Mann-Whitney *U* test was used to compare the difference between ultimate failure load, ultimate stress and stiffness of the repaired tendons. All statistical analyses were performed with the SPSS software (version 23.0; SPSS Inc., Chicago, IL, USA). The statistical significance was determined at *p* < 0.05.

## 3. Results

**Figure 3:**
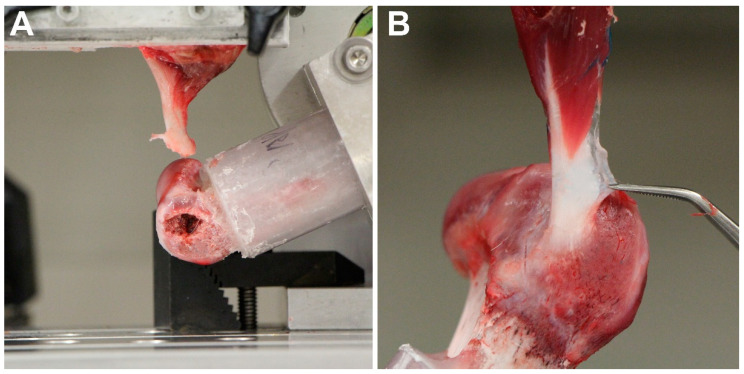
Representative site of failure for intact canine infraspinatus tendons in both groups. (**A**) Bony avulsion of the tendon from the greater tubercle at anatomic direction of pull. (**B**) Partial tendon tear near the tendon-bone interface at functional direction of pull.

No loosening of the humeral shaft or slippage of the tendon were observed throughout the biomechanical testing. The predominant failure mode of the intact tendon subjected to anatomic pull was bone avulsion of the tendon from the insertion on the humeral head (seven of ten specimens) (Figure 3A) (Table 1). In contrast, rupture of the tendon, either at or away from the tendon-bone interface, was seen for all intact specimens in the functional pull group (Figure 3B) (Table 1). With regard to the repaired tendons, four of eight specimens failed by suture break or suture pullout from tendon for the functional and anatomic pull group, respectively (Table 1).

**Figure 4:**
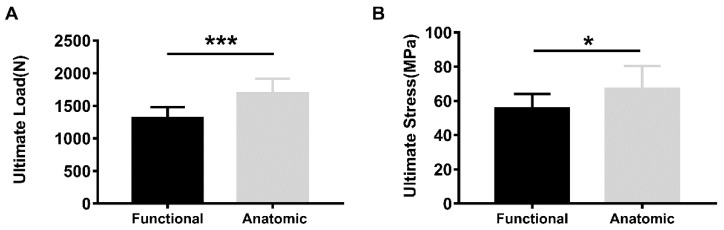
The ultimate failure load (**A**) and ultimate stress (**B**) of intact infraspinatus tendons under functional or anatomic pull (*n* = 10 per group) * *p* < 0.05, *** *p* ≤ 0.001.

**Figure 5:**
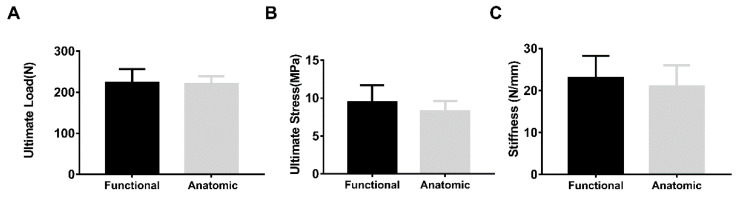
The ultimate failure load (**A**), ultimate stress (**B**) and stiffness (**C**) for infraspinatus tendons repaired in a modified Mason-Allen pattern under functional or anatomic pull (*n* = 8 per group).

When tested at the functional pulling angle, the intact infraspinatus tendon had a significantly lower mean ultimate failure load and ultimate stress than those of the anatomic pull group (1310.16 ± 167.62 N vs. 1687.39 ± 228.24 N, *p* = 0.0005: 55.64 ± 8.40 MPa vs. 67.13 ± 13.35 MPa, *p* = 0.0334) (Figure 4). There were no significant differences in the mean ultimate failure load, ultimate stress or stiffness between repaired infraspinatus tendons tested along the functional and anatomic direction (Figure 5). In addition, no significant difference was found in the mean cross-sectional area between groups for intact (functional vs. anatomic, 23.89 ± 3.93 mm^2^ vs. 25.52 ± 2.75 mm^2^, *p* = 0.2959) or repaired tendons (functional vs. anatomic, 24.15 ± 3.56 mm^2^ vs. 27.01 ± 2.99 mm^2^, *p* = 0.1038), respectively.

## 4. Discussion

The objective of this study was to determine whether the mechanical properties of intact tendon or tendon after repair is affected by the pulling angle set for mechanical testing in a canine shoulder model in vitro. The most important finding of our study was that intact canine infraspinatus tendon pulled at the functional angle demonstrated significantly lower ultimate failure load and ultimate stress compared to those pulled along the anatomic direction. In addition, we found that repair of the infraspinatus tendon using the modified Mason-Allen stitches resulted in comparable mechanical properties, including ultimate failure load, ultimate stress and stiffness, between functional and anatomic pull groups. These findings confirmed our hypothesis that different pulling angles do, in fact, have an effect on the rotator cuff biomechanics.

There are a variety of factors associated with rotator cuff healing and regeneration after surgical repair. In addition to providing a favorable biological environment that facilitates cell proliferation, growth factor regulation and collagen maturation, a suitable mechanical environment also plays a critical role in promoting the healing process and improving functional outcomes [37,38]. For example, Mihata et al. examined the retear rate after arthroscopic single-row, double-row and compression double-row rotator cuff repair which combines double-row and suture-bridge techniques. Based on magnetic resonance imaging, the overall retear rate was 10.8%, 26.1% and 4.7% for single-row, double-row and compression double-row repairs. Importantly, the retear rate of large and massive cuff tears was significantly reduced using the compression double-row technique, which provided enhanced compression of the tendon against the footprint compared to the other two techniques. Additionally, the lower retear rate was associated with better clinical outcomes at a minimum 24 months of follow-up [10]. In this regard, biomechanical testing has been extensively used in preclinical animal studies to evaluate the mechanical properties of repaired rotator cuff tendon. Although the selection of an animal shoulder model is primarily dependent on the research purpose, the mechanical testing modality differs among studies. When using smaller animal models, such as rat or rabbit, the rotator cuff tendon was usually tested at 90° of abduction. Rothrauff et al. used a 90° testing angle to investigate the effect of adipose-derived stem cells on enthesis healing in a rat supraspinatus tear model [17]. Yea et al. conducted the mechanical testing by positioning the rat shoulder at 90° abduction to evaluate the efficacy of umbilical cord-derived stem cells combined with a biomimetic scaffold on rotator cuff tendon-to-bone healing [28]. Of note is that a 90° angle between the tendon-bone interface and the supraspinatus tendon has been adopted to mimic the anatomic pull in both a rat and rabbit rotator cuff repair model [19,20]. Given the differences in rotator cuff structures between rat and rabbit, it seems that use of a common pulling angle would hardly replicate an anatomic pull in both of these animal models. Additionally, many authors did not specify the pulling angle but only described that the testing was along the functional or anatomic direction when using large animal models, such as dogs and sheep [15,21,22,24,25]. Interestingly, for the repaired infraspinatus tendon in sheep, the biomechanical testing could either be done by aligning the tendon to the humeral shaft at a 90° or 105° angle to simulate a functional or anatomic pull, respectively [26,29]. Thus, the interpretation and comparison of mechanical properties from various studies utilizing different animal models necessitates the standardization of the pulling angle.

To our knowledge, this is the first study to report the influence of pulling angle on the mechanical properties of rotator cuff tendon in a large preclinical animal model. In addition, our study compared the mechanical strength of the repaired tendon using the modified Mason-Allen pattern under functional or anatomic direction of pull. In 2016, Newton et al. assessed the effect of different abduction angles on the mechanical properties of the supraspinatus tendon in a rat model. The biomechanical testing was performed by positioning the rat shoulder at 0°, 30°, 60° and 90° of abduction. They found that the shoulder abduction angle was positively correlated with the tendon stiffness and modulus, and is associated with the crimp pattern and viscoelastic behavior change of the collagen fiber [32]. In our study, the intact canine infraspinatus tendon pulled at the functional angle exhibited lower ultimate failure load and ultimate stress than at the anatomic angle, which may predispose the tendon to injury. It has been suggested that collagen fiber realignment and crimp behavior can be affected by applied tensile loading [39,40]. Therefore, it is reasonable to postulate that all or most of the tendon fibers were recruited to bear tensile loading in the anatomic position, which led to failure at the bony site. However, in the functional position, only a portion of the tendon fibers participated in tensile load bearing with the remaining fibers maintaining a crimped status at the beginning of testing. This uneven load distribution on tendon fibers may result in a stress concentration on a group of fibers, thus leading to partial fiber damage or even rupture. Unlike bony failure at the greater tubercle for the anatomic group, the mode of failure for specimens tested along the functional direction was predominantly partial tendon tear at or away from the tendon-bone interface. This distinct failure mode further corroborated our assumption that functional pull would make the rotator cuff tendon more susceptible to tear, which resembles the clinical scenario that initial small or partial-thickness tears gradually progress to full-thickness tears, eventually leading to shoulder pain and dysfunction [41,42].

It is more important to note that the modified Mason-Allen technique yielded comparable mechanical strength in terms of ultimate failure load, ultimate stress and stiffness regardless of the pulling angle. Previous studies have shown that the modified Mason-Allen technique is a validated suture configuration for rotator cuff repair and often serves as a control for the evaluation of new suture material, suture configuration and postoperative rehabilitation [16,21,22,23]. Accordingly, we propose that for any novel treatment aiming to improve rotator cuff repair outcomes, the biomechanical testing in preclinical large animal models would be better performed using functional pull, which simulates the clinical setting to test its ability to prevent tear.

This study has several limitations. First, the specific angle defining a functional or anatomic pull varies among animal models. Although a few studies pointed out the specific angle simulating functional or anatomic pull, the majority of studies either did not mention the testing angle or simply described the pull direction. As canine shoulder is a widely used large animal model, our results would provide useful information on the supplementation and standardization of rotator cuff biomechanical testing protocols in preclinical animal studies. Second, we performed the testing on intact or acute full-thickness repaired infraspinatus tendon in vitro, which does not mimic the chronically injured and degenerated tendon repair situation in human patients. In addition, only time-zero mechanical properties were evaluated between the repaired groups. Future studies comparing the effect of pulling angle on enthesis healing in the context of a chronic tear in vivo are warranted. Third, the tendon repair was only performed in the modified Mason-Allen fashion; other clinically relevant repair techniques, such as single or double row with suture anchors, were not considered. However, the modified Mason-Allen technique with the use of 0-Fiberwire sutures is recommended for canine rotator cuff repair [21]. Finally, the collagen fiber realignment and viscoelastic behavior that might be responsible for the mechanical differences at varying pulling angles was not elucidated. Future studies are required to reveal the underlying fiber orientation and crimp patterns as a function of the pulling angle.

## 5. Conclusions

The variance of pulling angle had a significant influence on the biomechanical properties of the rotator cuff tendon in a canine shoulder model in vitro. Load to failure of the intact infraspinatus tendon was lower at the functional pulling position due to potential uneven load distribution on the tendon fibers compared to the anatomic pulling position. However, this mechanical character is not presented after rotator cuff repair using the modified Mason-Allen technique. In future studies, it would be interesting to investigate rotator cuff mechanical properties at varied shoulder abduction angles with human cadavers, which may reveal the shoulder position where the rotator cuff is most susceptible to injury.

## Figures and Tables

**Table 1:** Summary of the Failure Modes.

	Intact		Repaired	
Failure Modes	Functional (*n* = 10)	Anatomic (*n* = 10)	Functional (*n* = 8)	Anatomic (*n* = 8)
Bone avulsion at insertion	0	7	-	-
Proximal bone fracture	0	3	-	-
Soft tissue ^&^	10	0	-	-
Suture pullout from tendon	-	-	1	4
Suture pullout from bone	-	-	3	2
Suture break	-	-	4	2

^&^ Failure by tendon rupture away from or at the tendon-bone interface.

## Data Availability

The original contributions presented in the study are included in the article, further inquiries can be directed to the corresponding author.
